# Future Climate Scenarios for a Coastal Productive Planktonic Food Web Resulting in Microplankton Phenology Changes and Decreased Trophic Transfer Efficiency

**DOI:** 10.1371/journal.pone.0094388

**Published:** 2014-04-10

**Authors:** Albert Calbet, Andrey F. Sazhin, Jens C. Nejstgaard, Stella A. Berger, Zachary S. Tait, Lorena Olmos, Despoina Sousoni, Stamatina Isari, Rodrigo A. Martínez, Jean-Marie Bouquet, Eric M. Thompson, Ulf Båmstedt, Hans H. Jakobsen

**Affiliations:** 1 Institut de Ciències del Mar – CSIC, Barcelona, Spain; 2 P. P. Shirshov Institute of Oceanology RAS, Moscow, Russia; 3 Skidaway Institute of Oceanography, University of Georgia, Savannah, Georgia, United States of America; 4 Department of Biology, University of Bergen, Bergen, Norway; 5 Instituto de Investigaciones Marinas – CSIC, Vigo, Spain; 6 Department of Biology, University of Crete, Heraklion, Crete, Greece; 7 Institute of Oceanography, Hellenic Centre for Marine Research, Athens, Greece; 8 Sars International Centre, Uni Research, University of Bergen, Bergen, Norway; 9 Department of Ecology and Environmental Sciences, Umeå University, Umeå, Sweden; 10 Department of Bioscience, Aarhus University, Roskilde, Denmark; Stazione Zoologica Anton Dohrn, Naples, Italy

## Abstract

We studied the effects of future climate change scenarios on plankton communities of a Norwegian fjord using a mesocosm approach. After the spring bloom, natural plankton were enclosed and treated in duplicates with inorganic nutrients elevated to pre-bloom conditions (N, P, Si; eutrophication), lowering of 0.4 pH units (acidification), and rising 3°C temperature (warming). All nutrient-amended treatments resulted in phytoplankton blooms dominated by chain-forming diatoms, and reached 13–16 μg chlorophyll (chl) *a* l^−1^. In the control mesocosms, chl *a* remained below 1 μg l^−1^. Acidification and warming had contrasting effects on the phenology and bloom-dynamics of autotrophic and heterotrophic microplankton. Bacillariophyceae, prymnesiophyceae, cryptophyta, and *Protoperidinium* spp. peaked earlier at higher temperature and lower pH. Chlorophyta showed lower peak abundances with acidification, but higher peak abundances with increased temperature. The peak magnitude of autotrophic dinophyceae and ciliates was, on the other hand, lowered with combined warming and acidification. Over time, the plankton communities shifted from autotrophic phytoplankton blooms to a more heterotrophic system in all mesocosms, especially in the control unaltered mesocosms. The development of mass balance and proportion of heterotrophic/autotrophic biomass predict a shift towards a more autotrophic community and less-efficient food web transfer when temperature, nutrients and acidification are combined in a future climate-change scenario. We suggest that this result may be related to a lower food quality for microzooplankton under acidification and warming scenarios and to an increase of catabolic processes compared to anabolic ones at higher temperatures.

## Introduction

Global change may impact marine and freshwater plankton dynamics by altering many different factors and mechanisms. Three of them are believed to have particularly strong effects on aquatic ecosystems: eutrophication, ocean acidification, and warming [1]–[4].

Different future scenarios have been suggested for the distribution of nutrients in the upper mixed layer in coastal systems. Input and distribution of nutrients are often conditioned by the temperature-dependent physical stability of the water column. It has been argued that the predicted rise in temperature will increase the stability of the upper mixed layer of the open oceans and reduce primary production by decreasing available nutrients [5]–[7]. This oligotrophication of the ocean would favour small and flagellated cells compared to diatoms [8]. On the other hand, the phytoplankton production of coastal upwelling areas is predicted to increase because global warming may raise storm frequencies and wind intensities [5], [9]. In addition, increasing inorganic nutrient loads from human activities are threatening the stability of many coastal ecosystems [1] by impacting both the biomass and the size-structure of phytoplankton [10]. Increased upwelling and human-related discharges of nutrients, should be an advantage for larger phytoplankters (e.g., diatoms), and could ultimately result in increased planktonic food web productivity in coastal zones. Therefore, we hypothesize that *(i) inorganic nutrient additions (eutrophication) will result in a higher carrying capacity for all plankton, with higher expected biomasses compared to unaltered controls*. Assuming that enough dissolved inorganic carbon is available, any input of inorganic nutrients will result in enhanced phytoplankton production that will support higher grazer biomasses. However, *a marked reduction in trophic efficiency is expected under eutrophic conditions*, due to the limited capacity of grazers to use the boosted algae production [11]. Therefore, we expect (ii) *that the biomass of heterotrophs supported per unit biomass of autotrophs (as a measure of trophic efficiency,* [*12*] *) will be lower in the nutrient-enriched treatments*.

Ocean acidification, the second variable considered here, is a result of the decrease of the ocean's pH driven by the increased uptake of CO_2_ from the atmosphere [13]. This carbon-sequestering mechanism, which should serve to palliate global warming by reducing atmospheric CO_2_ concentrations, may impact oceanic biota. For instance, it has been shown that a decrease in pH has negative effects on marine calcifying organisms, including some phyto- and zooplankton, such as coccolithophores, foraminifera, larvae stages of corals, echinoderms, crustaceans and molluscs [14]–[16]. Laboratory and field experiments with non-calcifying unicellular organisms are less frequent and provide contrasting results [15]. In general, when the entire microbial community is analysed using bulk measures (e.g., chlorophyll), only modest or non-significant responses are observed [17]–[19]. Clear acidification effects on natural communities have been reported mostly on picoeukaryotes and some phototrophic bacteria [20]–[22]. These results contrast with species-based observations showing that different species, or even strains (e.g., the coccolithophore *Emiliania huxleyi*; [14], [23]) responded in different ways to acidification [18], [19]. We believe that a number of species-specific responses occur in field experiments (and nature), although they may pass undetected because of insufficient analytical and/or taxonomic resolution in field experiments. Globally, increasing CO_2_ implies more carbon availability for phytoplankton. We therefore hypothesize; that *(iii) increased partial pressures of CO_2_ in nutrient-rich waters will result in higher phytoplankton primary production* [*2*] *, but will be disadvantageous for specific calcifying groups, such as coccolithophores*. The extra autotrophic biomass, however, may be of poorer food quality [2], [24], [25]. Therefore, we hypothesize that *(iv) the growth response of the protozoan grazers may not be proportional to the surplus food availability.*

The last variable considered here, temperature, controls all physiological rates. For each species, there is an optimum temperature (Arrhenius break temperature, ABT) for the different anabolic and catabolic processes, above which, the rate of the process declines. Different ABT for catabolism and anabolism will result in different efficiencies of growth for protists at different temperatures. Literature reviews and syntheses point toward equivalent, or even higher, maximal growth rates of herbivorous protozoans compared to phytoplankton at temperatures above 15°C [26]. This experimental study covers temperatures both above and below 15°C, thus the difference in growth rates of heterotrophs *vs* phytoplankton should be more pronounced [27], [28]. Consequently, it could be hypothesized that *(v) an increase in temperature will accelerate the onset and breakdown of the phytoplankton bloom* by a combined effect of higher growth rate of phytoplankton (faster inorganic nutrient consumption) and an enhanced grazing impact of protozoa [26]. Conversely, a negative relationship of protozoa gross growth efficiency (GGE) and temperature leads to less-efficient food conversion into growth at higher temperatures [29]. Further, because high algal growth rates show an unbalanced investment in N and C [30] algae have lower nutritional quality *i.e*. higher C:N ratios at higher temperatures. We therefore hypothesize that (*vi*) growth of *protozoans will be negatively impacted by increased temperature*, and thus *(vii) future predicted warming conditions should lower the overall food web transfer efficiency of matter and energy from the primary producers to higher trophic levels*.

Here, we assess the effects of different global change scenarios on the trophic efficiency of a coastal food web by analysing the numerical, biomass (e.g., ratio heterotrophs/autotrophs), and stoichiometric responses of the planktonic protist community. We simulated possible future synergistic climate conditions by manipulating nutrients, temperature, and pH. The contemplated scenarios were i) unaltered communities, ii) eutrophication, iii) eutrophication combined with acidification, and iv) eutrophication combined with acidification and warming. This was conducted in a mesocosm experiment with enclosed natural plankton in outdoor tanks. We used modelled values from IPCC for the end of century, which predict a 3°C rise of temperature and a decrease of 0.4 pH units compared to the present levels [31]. Although the effects of these variables have been studied independently on single species and marine communities, they have seldom been considered together [32]–[35]. Synergic effects may boost the response of organisms to a higher extent than the sum of all responses to each variable alone.

## Methods

### Experimental set-up and physical-chemical variables determination

We conducted a land-based mesocosm experiment at the Marine Biological Field Station of the University of Bergen, Norway from June 10^th^ to 30^th^, 2011. The experiment included 4 treatments (control, +eutrophication, +acidification, and +warming; Table 1) in duplicate (A and B; for phytoplankton taxonomy, only replicate A was analysed). This study was a part of a larger experiment hosted by the Nordic Council project BIOPUMP, the EU FP7 project MESOAQUA and a Norwegian Ocean and Coast research grant (see financial disclosure). The light-regime during the mesocosm experiment displayed a 19∶5 day:night cycle with a maximum light intensity on air of ca. 300000 Lux (ca. 5500 μmol photon m^−2^ sec^−1^) measured by HOBO units (Onset Computer Corporation, Bourne, MA, USA). The initial (day 0) underwater light climate within the mesocosms measured at 1 m depths did not show difference between the treatments (ANOVA, p = 0.55).

**Table 1:** Experimental design and initial conditions for the different treatments.

		Treatments		
Symbol/Line	Mesocosms	Nutrients	pH	Temperature
Filled square-dashed line	M1	Ambient	ambient	Ambient
Open square-solid line	M2	high	ambient	Ambient
Filled circle-dashed line	M3	high	low	Ambient
Open circle-dotted line	M4	high	low	High

Mesocosms M1 were controls with natural ambient nutrients, ambient pH (8.15), and temperature (12.3°C). In M2, M3, and M4 8 μM NaNO_3_, 0.5 μM K_2_HPO_4_ and 5 μM Na_2_SiO_3_ were added at the beginning of the experiment. In mesocosms M3 and M4, the CO_2_ concentrations were increased from ca. 400 to 1000 ppm (pH from ca. 8.15 to ca. 7.7). The pH of M2 was also controlled to avoid excessive alkalization as a result of the activity of the algae during bloom conditions and was kept similar to M1. In mesocosms M4, the temperature was raised to a target of ca. 3°C above ambient with an actual attained difference of 2.7°C. The effects of the climatic drivers can be detected by comparing sets of mesocosms with each other: The effect of nutrients by comparing M1 with M2, the effect of pH by comparing M2 with M3, and the effect of temperature by comparing M3 with M4.

Experiments were initiated on June 10^th^ (day -1) by filling unfiltered, nutrient-poor seawater into eight, 2.5 m^3^ fiberglass mesocosms, which were open to the atmosphere. The tanks were filled using a plankton pump (Unik Filtersystem, Oslo, Norway) in staggered mode so all mesocosms had equal starting conditions. Filling rates ranged from ca. 300 to 500 L min^-1^ depending on the elevation over the sea of the mesocosms. The mesocosms were placed in larger tanks that received continuous water flow from the fjord to maintain the mesocosms at approximately *in situ* temperature. Each of the larger tanks was equipped with two bilge pumps to keep the temperature even in the larger tanks. The temperature increase in mesocosm M4 was established by warming the water in one of the larger tanks with a commercial pool heating device consisting of a 6 kW electrical heating unit combined with a circulation pump (Saci 0.37 kW WINNER, 50 M 230 V mono phase, mounted on a 50 mm PVC tube, Pahlén Norge AS, Billingstad, Norway, and combined with a SAAS A/S temperature regulation system, Oslo, Norway). The temperature in the tanks was gradually increased during the first day avoiding abrupt changes. We measured and controlled the temperature in the outer pools during the experiment using a portable Multi-parameter probe WTW Multi 3420 twice a day (data not shown). In addition, the temperatures were continuously logged every 10 min throughout the experiment at 1 m depths in the mesocosms and the outer pools using HOBO units (Onset Computer Corporation, Bourne, MA, USA).

The pH was regulated by addition of gaseous CO_2_ using a computerized AquaMedic control system (AB Aqua medic GmbH, Germany). The pH gradually stabilized within the first day. The water inside the mesocosms was gently mixed by slow bubbling of large (>5 cm) bubbles released at the bottom of the mesocosm every 3–5 s. This method has been successfully developed to minimize destruction of delicate plankton, while resulting in sufficient mixing as described in detail by Troedsson et al. [35]. All the treatments including inorganic nutrient addition, temperature and pH manipulation were initiated on day 0. The pH of all mesocosms was measured twice a day with the portable Multi-parameter instrument WTW Multi 3420 equipped with a WTW Sentix 980 pH probe, calibrated with National Bureau of Standards (nbs) buffers (Hamilton calibration buffer) and Total Scale (TS) buffers following Dickson et al. [36]. Mesocosms M1 and M2 without addition of CO_2_ were pH regulated as well to avoid unrealistic elevations of pH as a result of the artificial enhanced phytoplankton production.

For nutrient determination, approximately 20 ml of water sample was filled up in a syringe (BD PlastipakTM Luer-Lok Syringe 20 ml) and then connected to a syringe filter (Acrodisc 32 mm Syringe Filter with 0.2 μm Supor Membrane, sterile), which had been pre-washed two times in distilled water. The first 7–8 ml of filtered water was used to rinse the sample tube (BD 15 ml High-Clarity Polypropylene conical Falcon tubes), and the sample tube was then emptied and finally filled up with 10–11 ml filtered water, avoiding any air to pass the filter. The procedure was repeated for each sample, using a new filter each time. Blanks were prepared as described above, using synthetic seawater. The samples were immediately frozen at −20°C and stored for two months prior analysis. Dissolved nitrate, phosphate and silicate were analyzed in duplicate from the thawed samples, using a 4-channel auto-analyzer (QuAAtro marine, Bran & Luebbe) according to the Swedish standards Institute and HELCOM [37]. Extended measurement uncertainty varies between 8.4 and 22% depending on substance.

### Community composition and biomass

Every day 100 ml water samples were collected from the mid part of the mesocosms at approximately 1 m depth to determine chlorophyll *a* (chl *a*) concentrations in each mesocosm. The water was sequentially filtered onto 10, 2, 0.6, and 0.2 μm pore-sized polycarbonate filters of 47 mm diameter (GE Water & Process Technologies) to obtain the size-fractions: 0.2–0.6 μm, 0.6–2 μm, 2–10 μm, and >10 μm, respectively. The filters were extracted immediately in 90% acetone overnight at −20°C and measured on a 10-AU Turner fluorometer (Turner Designs, Sunnyvale, CA) according to Parsons et al. [38].

Water samples of 50–100 ml were taken for microscopy of the phytoplankton communities in replicate A of each mesocosm treatment on days 0, 1, 2, 4, 6, 8, 11, and 14. These samples were stained with primuline (Direct Yellow 59, Sigma-Aldrich Co), fixed with 3.6% glutaraldehyde solution with 10% glycerol (final concentrations), and gently filtered onto black 25 mm diameter 0.6-μm pore-size polycarbonate membrane filters, mounted on slides and frozen at −20°C until analysis by epifluorescence microscopy within 3 days of sampling. The method is a modification from Grebecki [39], Hobbie et al. [40] and Caron [41] with the glycerol added to reduce the damage of especially small delicate protists during filtration as described in Sazhin et al. [42]. Cell volumes were calculated by approximation of simple geometrical 3D shapes and converted into cell carbon as described in Menden-Deuer and Lessard [43]. The data on carbon biomass in replicate A of each mesocosm were used to calculate a C:chl *a* ratio that was then applied to the replicate B. By this means we obtained replicated carbon values of autotrophs to be compared with those of protozoan microplankton (basically, *Gyrodinium spirale* and ciliates; see below).

Sample preservation may result in considerable loss of larger delicate protists [44]. Therefore, we also analysed samples of untreated live plankton from both replicates of each mesocosm using two black and white camera FlowCAM II instruments (http://www.fluidimaging.com/). The FlowCAMs were run in autoimage-mode, using 4x magnification to analyse particles ranging between 15 and 1000 μm, focusing on ciliates and the dominant athecate dinoflagellate *Gyrodinium spirale*. The samples were kept in dim light at 12°C until analysed within 4 hours after sampling. Each sample was run for ca. 30 min, corresponding to 5.7 ml of analysed volume. The context capture properties chosen to do the analysis were: distance to the nearest neighbour 20 μm; close holes iteration 4; convolution filter smooth; collage image border padding 4 pixels; particles were defined by dark pixels with segmentation threshold of 12 pixels. All the image collages were post analysed in order to separate the particles in question (ciliates and *Gyrodinium spirale*). Particle sizes were determined from area-based diameter [45] and converted into biomass using the equations provided in Menden-Deuer and Lessard [43].

To determine the organic particulate fractions of carbon and nitrogen (POC and PON) 0.5–2 L water samples were pre-filtered at low vacuum pressure through 10 μm polycarbonate filters (GE Water & Technologies, Manchester, UK) and filtered onto pre-combusted glass fiber filters (GF/D, 25 mm diameter, 2.7 μm pore size, Whatman). The GF/D filters were transferred into clean petri dishes, dried overnight at 65°C and stored frozen at −20°C until analysis. Particulate carbon and nitrogen amounts per filter were measured using a Flash EA elemental analyzer coupled to a ThermoFisher Delta V plus Mass Spectrometer. Carbon: Nitrogen (C:N) molar ratios were then calculated for the size fraction of 2.7–10 μm, representing the size of the protozoan prey.

The software package Prism 5.0 was used to conduct the statistical tests. The significance threshold used was 0.05, unless indicated. The tests used included two-way grouped ANOVA with repeated measures and Bonferroni *post hoc* test, forward stepwise multiple linear regression, and ANCOVA.

## Results

### Temperature and pH

**Figure 1:**
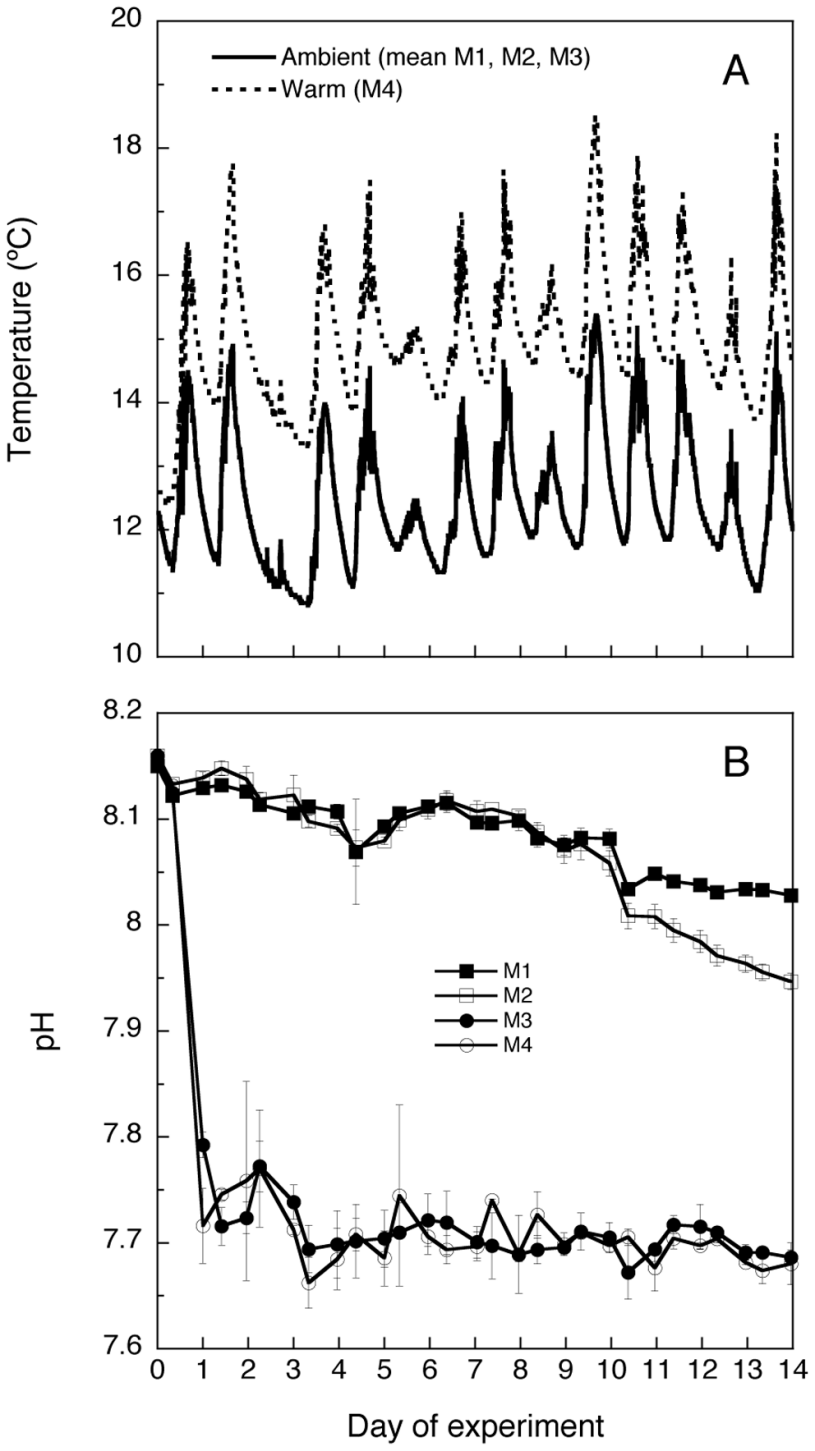
Temperature and pH. Variation of temperature (A) and pH (B) in the four mesocosm treatments (M1-M4, see Table 1 for explanation) throughout the experiment. Temperature values logged every 10 min at 1 m depth using HOBO units have been averaged for the ambient temperature treatments (M1-M3), while two daily pH measurements have been provided for each separate mesocosm treatment. Replicated treatments (A, B) for each treatment have been averaged in each case and errors bars correspond to SD.

We obtained an average temperature difference of +2.7°C in the warm *vs*. the ambient temperature treatments and a ca. 0.4 pH units decrease in the low *vs*. ambient pH treatments (Fig. 1A, B). No detectable temperature difference between the surface and the bottom of the mesocosms indicated a well-mixed system (data not shown). The temperature variation between day and night was 1 to 4°C in all mesocosms (Fig. 1A). During the experiment, pH slightly decreased from 8.16 to 8.04 in the ambient pH mesocosm M1 (Fig. 1B). Mesocosm M2 was maintained at a similar pH to M1 during most of the period. However, towards the end of the experiment (from day 10 on), M2 slightly decreased in pH to 7.94. The low pH treatments remained constant around 7.7 in mesocosms M3 and M4 (Fig. 1B).

### Inorganic nutrients

**Figure 2:**
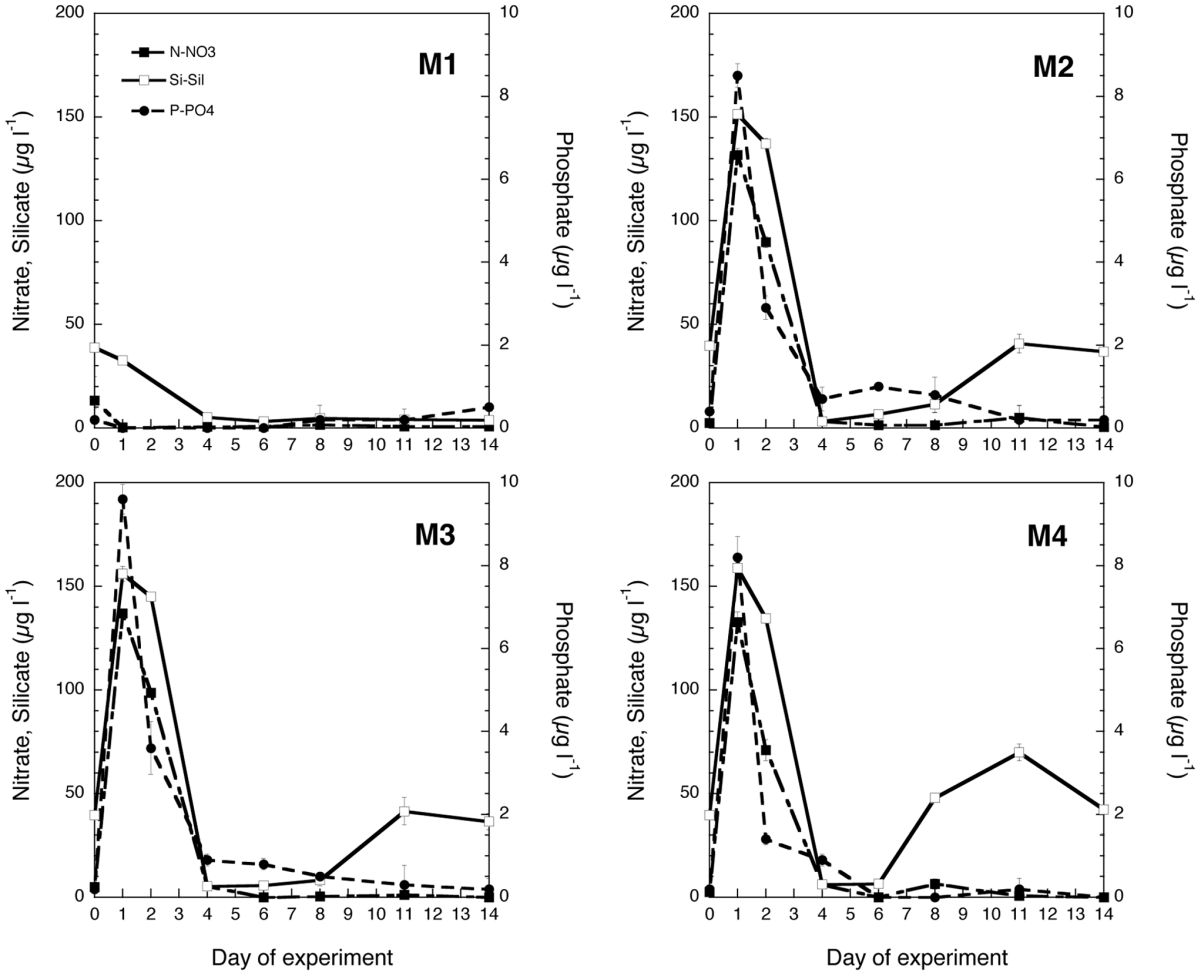
Inorganic nutrient variation in the four mesocosm treatments (M1-M4) throughout the experiment: A) nitrate B) phosphate C) silicate. Values are daily averages of the two replicates of each treatment and bars indicate the SD.

Inorganic nutrient amendment treatments were ca. 4 times the original values for silicate, and 20–50 times for nitrogen and phosphorous, respectively (Fig. 2). Despite these initial high concentrations, the dissolved nutrients were taken up rapidly, and after 4 days the concentration levels were comparable to the initial low values (Fig. 2). The only recycling episode evident was for silicate, which showed a secondary peak around day 11 in all nutrient amended mesocosms, the most conspicuous being in M4 (Fig. 2C).

### Chlorophyll a

**Figure 3:**
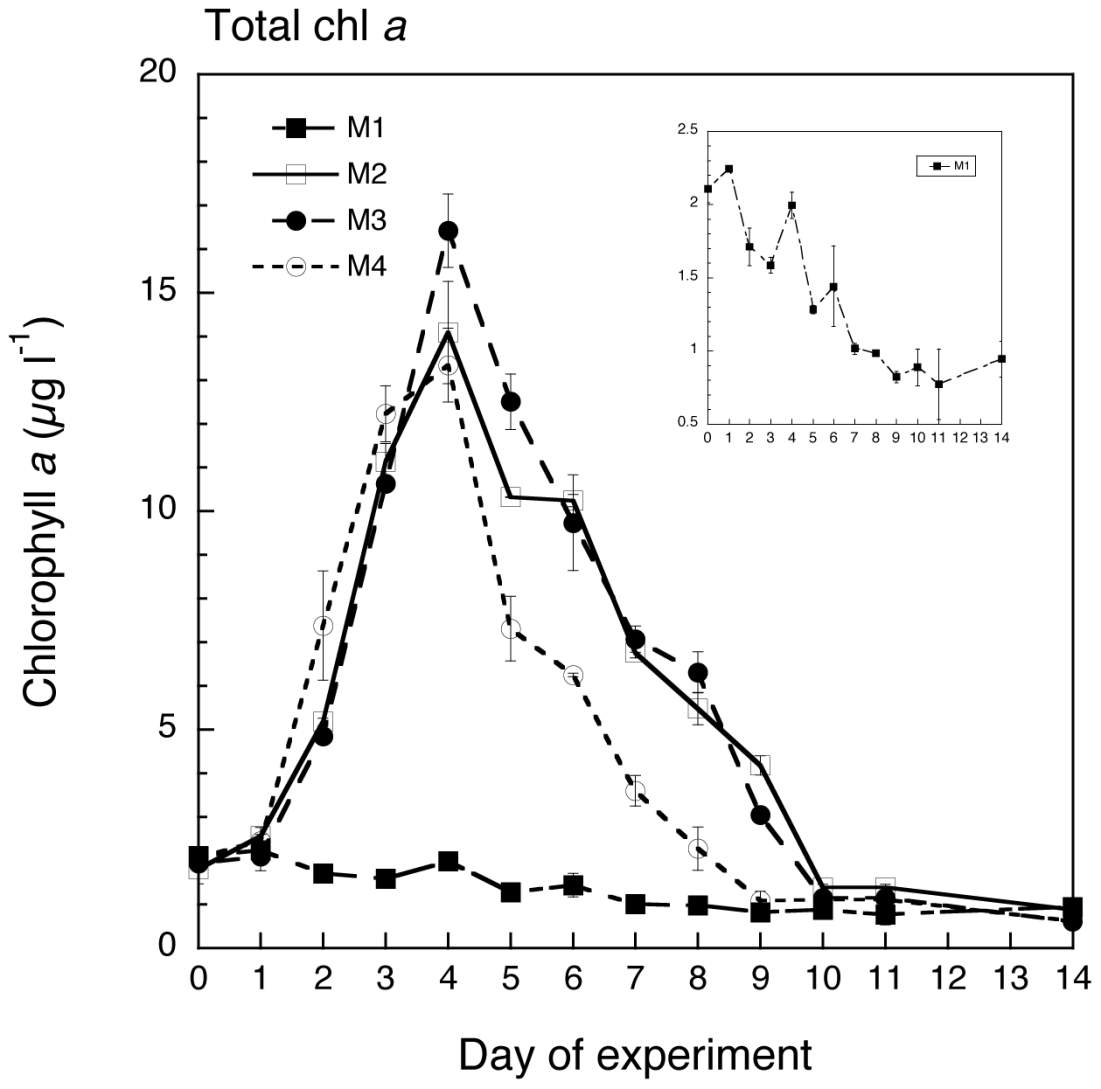
Temporal variation of chl *a* in the four mesocosm treatments (M1-M4) throughout the experiment. Values are daily averages of the two replicates of each treatment and bars indicate the SD. Note that values of the control mesocosm (M1) have been also presented separately in an inner plot to allow comparison with the notably higher values of the other three treatments.

The addition of nutrients resulted in clear bloom dynamics in all nutrient amended mesocosms (M2, M3, M4), whereas the chl *a* concentrations in the control (M1) remained low and declined from 2.2 to below 1 μg chl *a* l^−1^ (Fig. 3; Table 2). The highest chl *a* concentrations (ca. 16 μg L^−1^) were observed in the M3 mesocosms (high nutrients, low pH and ambient temperature), indicating that pH had a significant effect on the magnitude of the peak (Table 2; two-way grouped ANOVA with repeated measures and Bonferroni *post hoc* test). This effect was mostly produced by >10 μm chl *a*. Mesocosms M2 and M4 showed similar maximal concentrations around 13–14 μg L^−1^ chl *a*. However, the temporal pattern was rather different (Fig. 3). Although the mesocosms with higher temperatures (M4) peaked at the same time as the other nutrient-amended mesocosms, M4 had an earlier onset and also showed a faster decline of the phytoplankton bloom (ca. 2 days faster) indicating a significant temperature effect on the timing of the phytoplankton bloom (Table 2). Again, these differences are mostly driven by >10 μm chl *a* (Fig. 3, Table 2).

**Table 2:** Results of a two-way grouped ANOVA with repeated measures.

	Nutrients	Acidification	Temperature
**Total chl ** ***a***	*** (days 2–9)	ns (days 4,5)	* (days 2, 6–8)
**>10 μm chl ** ***a***	*** (days 2–9)	ns (day 4)	** (days 4–9)
**3–10 μm chl ** ***a***	** (days 2-5, 7)	ns	ns
**1–3 μm chl ** ***a***	** (days 2–7)	ns	ns
**0.2–1 μm chl ** ***a***	* (days 2–5)	ns	ns

The significance of the effect of the factors nutrients, acidification, and temperature has been contrasted against their respective controls for the different size-fractions of chlorophyll a (i.e., Nutrients: M2 vs M1; Acidification M3 vs M2; Temperature: M4 vs M3). Significance level for the entire period is indicated with asterisks for each size-fraction. Moreover, significant differences between treatments at specific days (Bonferroni post *hoc* test) at p<0.05 are also indicated. Note that a variable may not be significantly different from its control for the entire period, but showing significant differences at specific dates.

ns = not significant; * = p<0.05; ** = p<0.01; *** = p<0.001.

**Figure 4:**
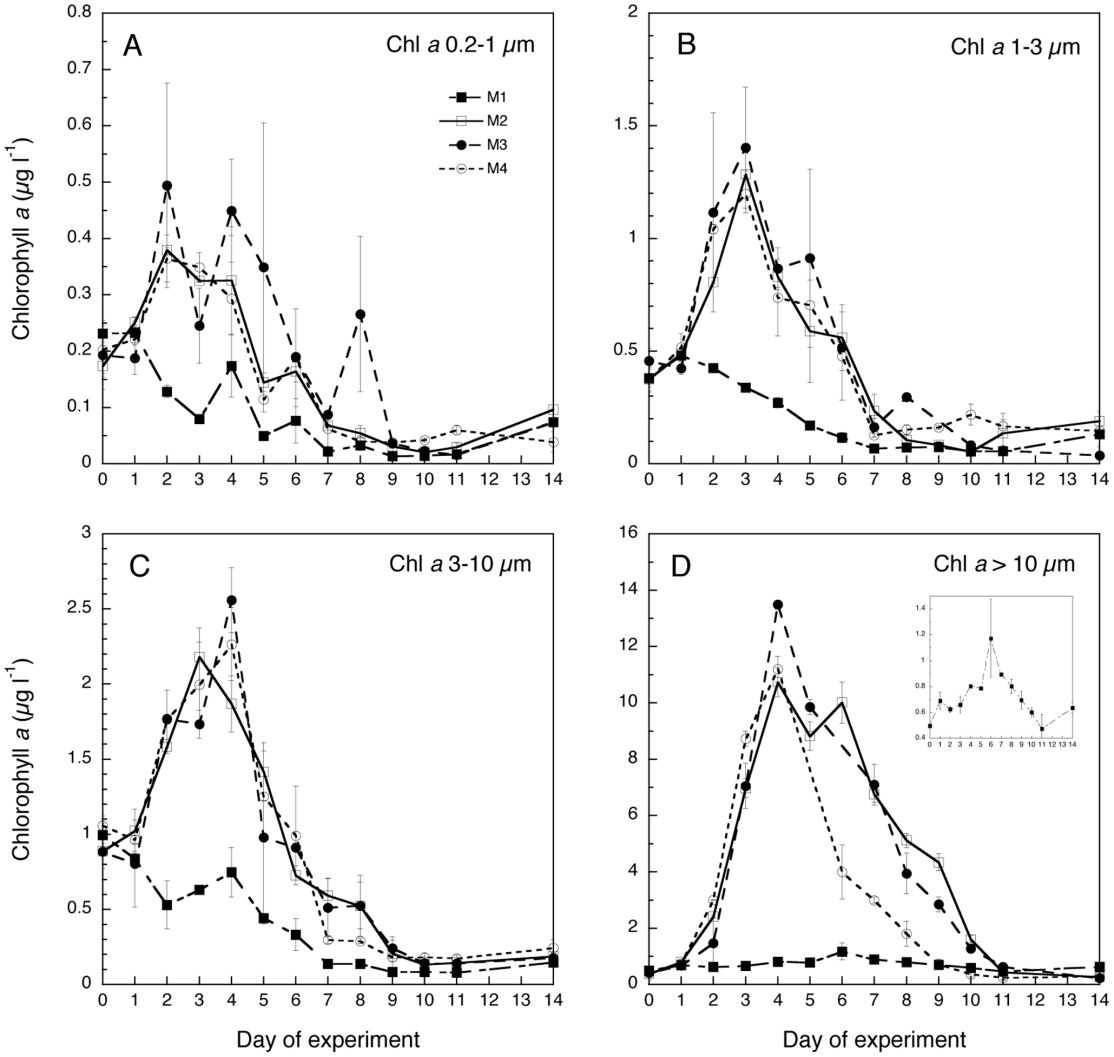
Temporal variation of the size fractionated chl *a* concentration in the four mesocosm treatments (M1-M4): A) 0.2–1 μm B) 1–3 μm C) 3–10 μm D)>10 μm. Daily averages of the two replicates per treatment have been provided and bars indicate the SD. Note that values of the control mesocosm (M1) have been also presented separately to allow comparison with the notably higher values of the other three treatments.

At the start of the experiment, phytoplankton size distributions were similar in all mesocosms and dominated by the 3–10 μm size-fraction (Fig. 4). In the unfertilized M1 mesocosms, the >10 μm fraction stayed relatively unchanged, while all smaller fractions decreased slightly during the experiment. In the fertilized mesocosms (M2-4) all fractions increased initially, with the highest values reached in the >10 μm fraction on day 4. During this bloom period there was a gradual shift toward dominance of larger algae with the largest fraction (>10 μm) surpassing the chl *a* of all smaller fractions together during the bloom. Mesocosms M3 showed the highest peaks in chl *a* for both the >10 and 3–10 μm fractions. While M4 showed an earlier decrease in chl *a* >10 μm compared to the other fertilized mesocosms, M3 and M4 indicated a later peak (day 4) in the 3–10 μm fraction compared to M2 (day 3). From day 11 to 14, all mesocosms, including the untreated M1, showed similarly low chl *a* concentrations in all fractions.

### Major plankton groups from microscopic counts

**Figure 5:**
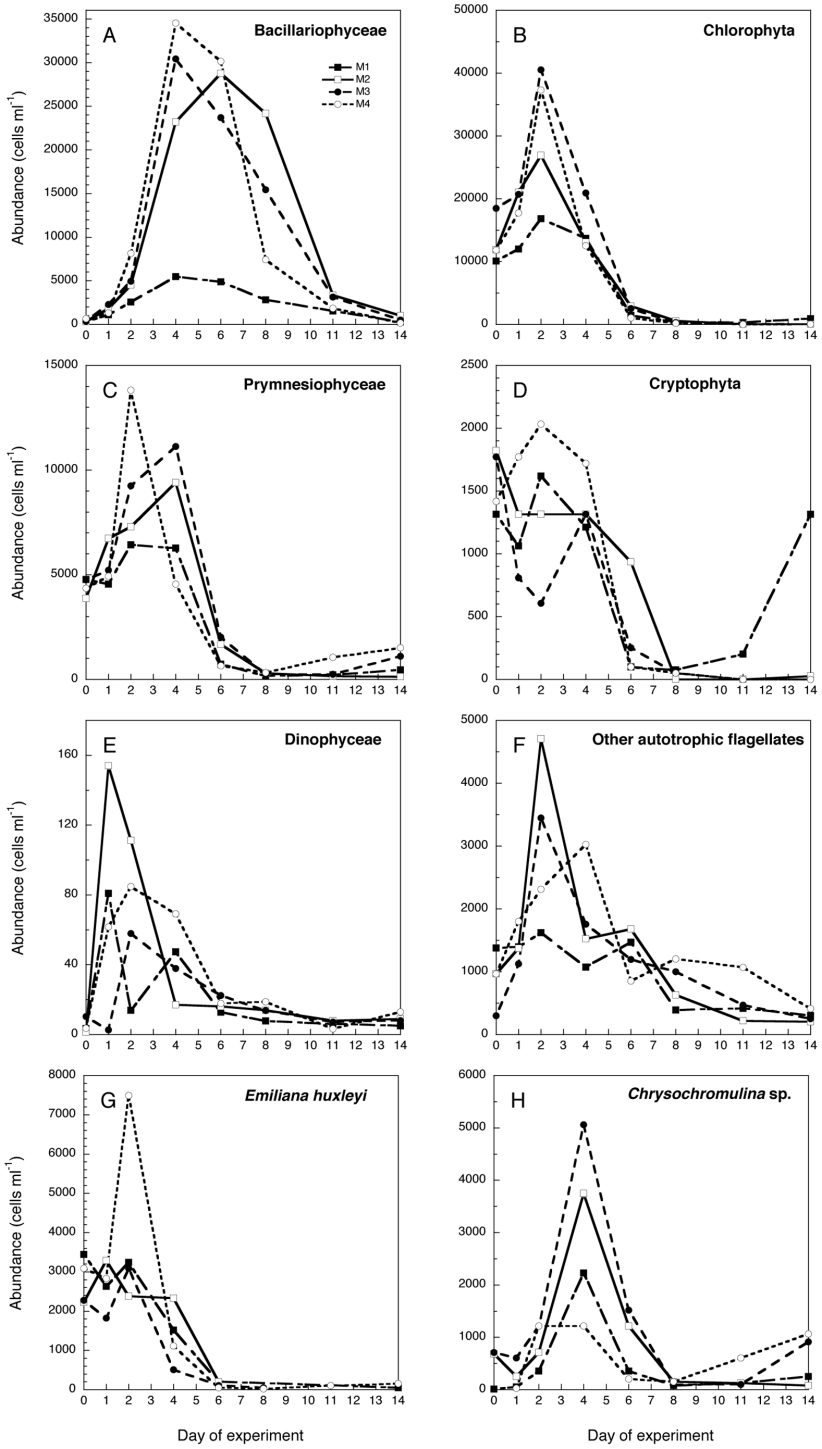
Concentration of the major autotrophic groups: Bacillariophyceae, Chlorophyta, Prymensiophyceae, Cryptophyta, Dinphyceae, and autotrophic flagellates in the four mesocosm treatments (M1-M4) throughout the experiment. Note that values correspond to the replicate A of each mesocosm treatment.

**Figure 6:**
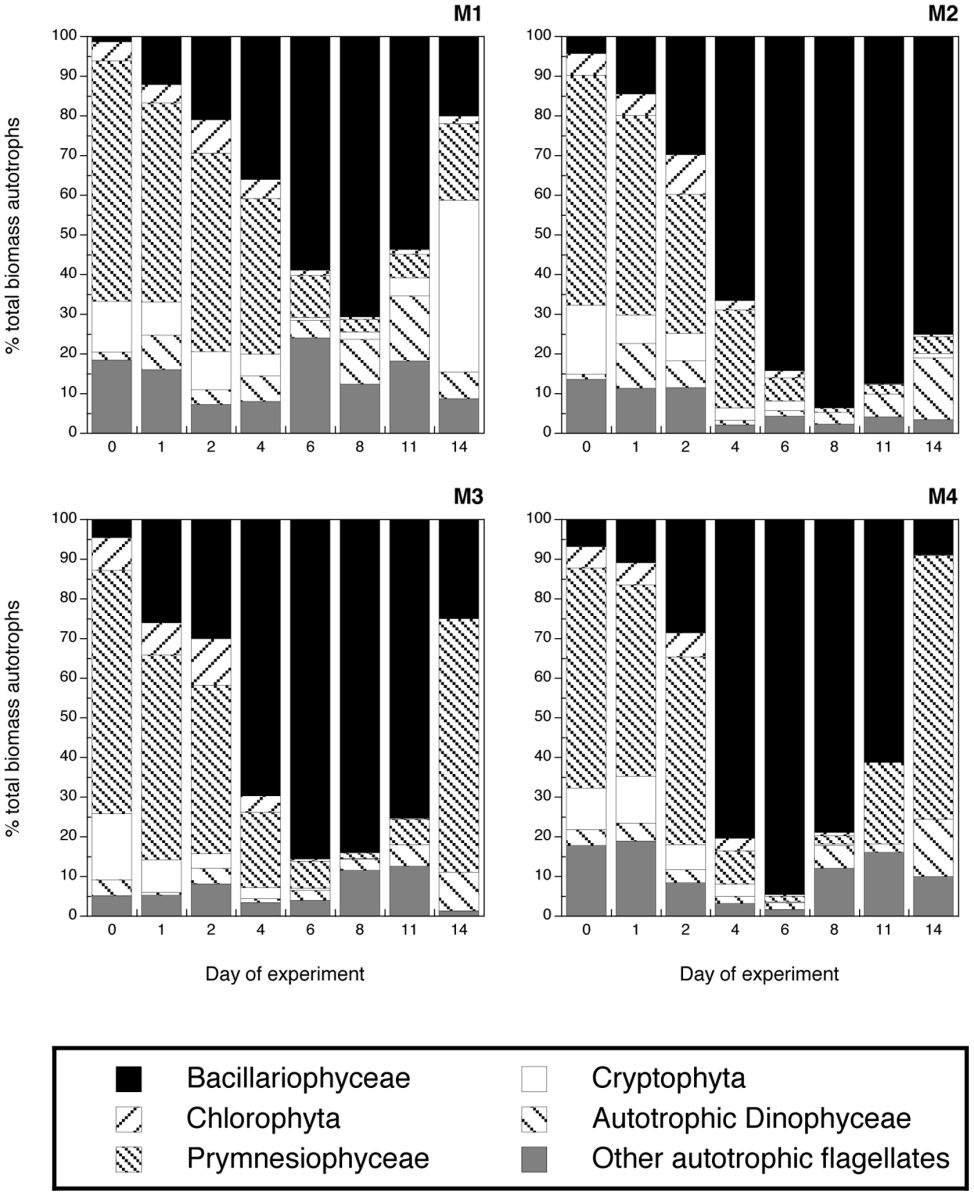
Biomass contribution of the major autotrophic groups to the total autotrophic biomass: Bacillariophyceae, Chlorophyta, Prymensiophyceae, Cryptophyta, Dinphyceae, autotrophic flagellates. Note that values correspond to the replicate A of each mesocosm treatment.

Bacillariophyceae (Fig. 5A): Diatoms did not contribute substantially to the initial phytoplankton of the fjord (Figs 5A and 6); however, diatoms responded swiftly and peaked numerically on day 4 in M1, M3, and M4, and decreased rapidly thereafter in these mesocosms, fastest in M4. M2 showed both a later peak (day 6) and a later onset of the most rapid decline (day 8) compared to the other mesocosms (Fig. 5A). Comparing the development of the diatom numeric abundance (Fig. 5A) and the nutrients (Fig. 2), shows that the diatoms arrested their growth when nutrients were exhausted but remained in the water column in all treatments a few days after nutrients were depleted.

Diatoms became the dominant group (mostly *Skeletonema marinoi*) in terms of biomass from day 4 in the nutrient amended mesocosms and from day 6 in M1 (Fig. 6). By the end of the experiment diatoms still dominated the community in terms of biomass in M2, but not in the other mesocosms, where Prymnesiophyceae (M3 and M4) and Cryptophyceae (M1) contributed predominantly to the autotrophic biomass after day 11.

Chlorophyta (Fig. 5B): A very short and pronounced peak at day 2 defined this group followed by a decline of the abundances to become almost absent from the community from day 6 onwards (Figs. 5B and 6). Differences were evident in the peak magnitudes, where the acidified treatments displayed the highest values. The highest contributor to the community of chlorophyta was the pico-eukaryote *Micromonas pusilla*.

Prymnesiophyceae (Figs. 5C, G and H): The most-abundant species within the group was *Phaeocystis pouchetii*, followed by *Emiliania huxleyi* and *Chrysochromulina alifera*. The behaviour of the Prymnesiophyceae was different in M4 compared to the rest of mesocosms (Figs. 5 and 6). The peak in the warm treatment M4 for these algae was more pronounced and occurred earlier, already on day 2 with a large contribution of *E. huxleyi* (Fig. 5G), while all colder mesocosms (M1-3) peaked on day 4 with a much larger dominance of *Chrysochromulina* spp., especially in the acidified M3 (Fig. 5H). From day 6, the biomass contribution of the Prymnesiophytes was low in all mesocosms, until the last day of the experiment when they again dominated the community biomass in both M3 and M4, mainly due to a second increase in *Chrysochromulina* spp. (Fig. 6). Thus, *E. huxley* displayed an early positive response to temperature, whereas the opposite pattern was the case for *Chrysochromulina* spp., dominated by *C. alifera*. In addition *Chrysochromulina* spp. showed a positive response in both the mesocosms with increased CO_2_ levels (M3 and M4) at the end of the experiment.

Cryptophyta (Fig. 5D): The peak of this group was negatively affected by acidification and positively by temperature (Figs. 5D, 6). The abundances declined in all treatments after day 4. However, there was a secondary peak in M1 on day 14, where cryptophytes ended as the dominant group in terms of biomass (Fig. 5D, 6). *Plagioselmis prolonga* was the only important contributor to the group.

Dinophyceae (Fig. 5E): Autotrophic dinoflagellates were modest contributors to the phytoplankton community (Fig. 6). The most-pronounced peak was observed in mesocosm M2 on day 1, and lower peaks of M1 and M3, M4 were observed on day 1 and 3 respectively. After day 6 the abundance in most of the mesocosms had fallen to less than 20 cells ml^−1^ (Fig. 5E).

Other autotrophic flagellates (Fig. 5F): The highest peak abundance was observed in M2, while mesocosms M3 and M4 peaked at lower abundances on day 2 and 4, respectively. No distinct peak was evident for other autotrophs in M1. After day 4, the abundances slowly decreased in all mesocosms until the end of the experiment (Fig. 5F).

**Figure 7:**
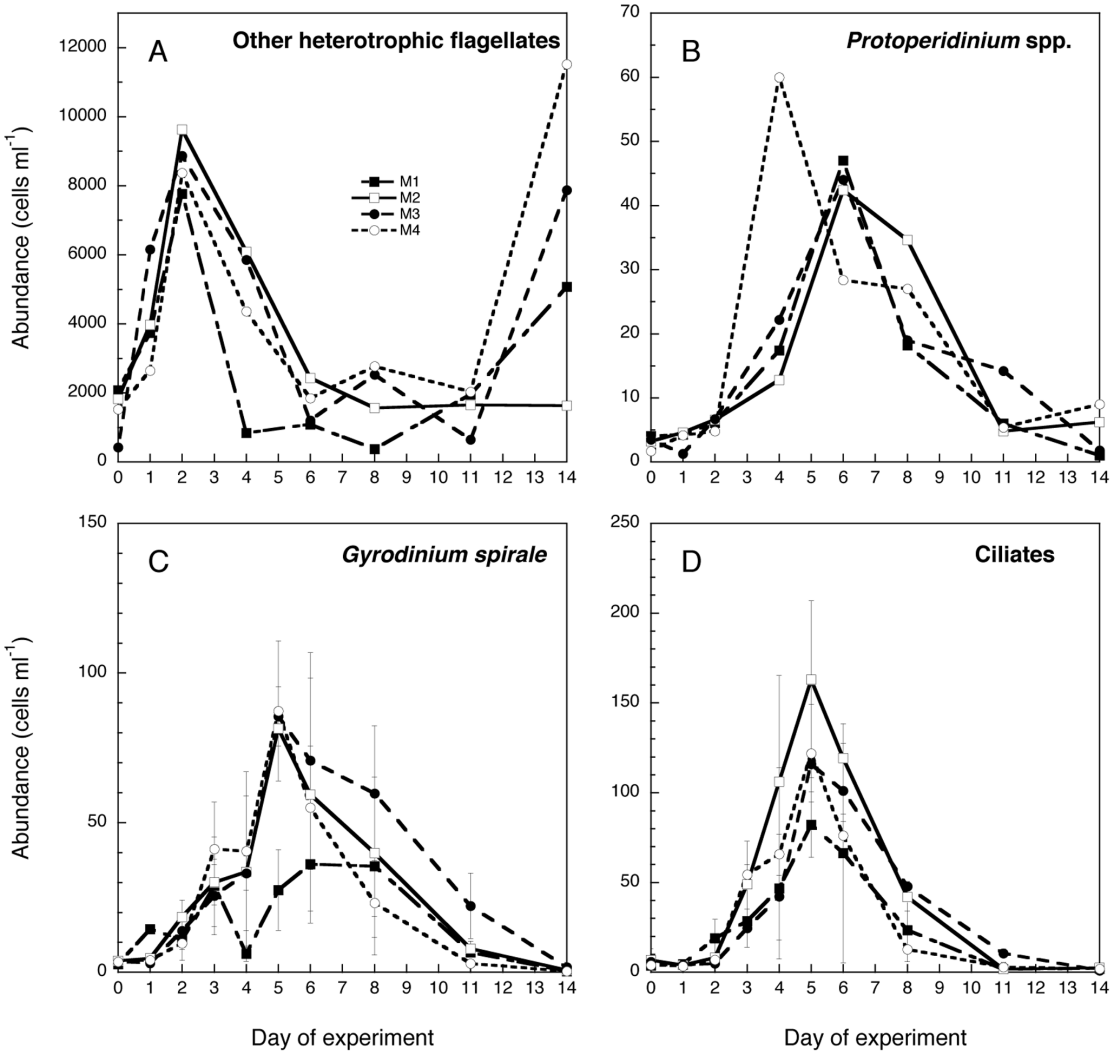
Concentration of the major heterotrophic groups in the four mesocosm treatments (M1-M4): Heterotrophic flagellates, *Protoperidinium* spp., *Gyrodinium spirale*, and ciliates in the four Note that the values of heterotrophic flagellates and *Protoperidinium* spp. correspond to microscopic counts for the replicate A of each mesocosm treatment. Abundance of *Gyrodinium spirale* and ciliates are averages of both replicates (A, B) of each treatment assessed by the FlowCam. Error bars indicate SD.

**Figure 8:**
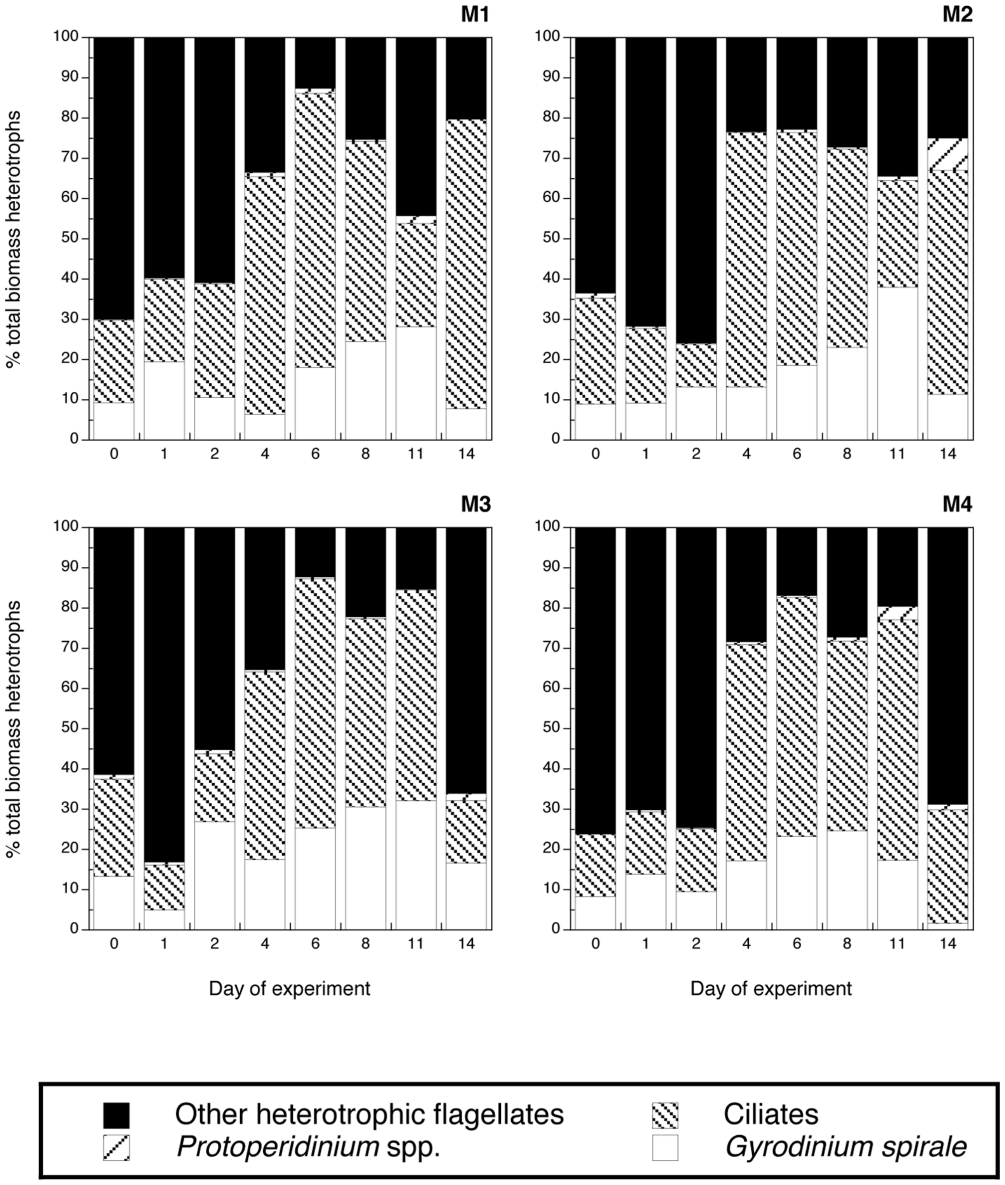
Contribution of the major heterotrophic groups to the total heterotrophic biomass in the four mesocosm treatments (M1-M4): Heterotrophic flagellates, *Protoperidinium* spp., *Gyrodinium spirale*, and ciliates. Note that the values of heterotrophic flagellates and *Protoperidinium* spp. correspond to microscopic counts for the replicate A of each mesocosm treatment. Abundance of *Gyrodinium spirale* and ciliates are averages of both replicates (A, B) of each treatment assessed by the FlowCam.

Other heterotrophic flagellates (Fig. 7A): Unidentified flagellates numerically dominated the heterotrophic community. They peaked in all mesocosms on day 2 at similar numbers, but thereafter they decreased in abundance in all mesocosms and stayed low in M2, while they increased again at the end of the experiment in M1, M3, and M4 (Fig. 7A). The most-pronounced increase at the end of the experiment was in the warm and acidified treatment (M4), and second-highest in the acidified treatment only M3 (Fig. 7A). In terms of carbon they were most important relative contributors of the heterotrophic biomass initially until the second day of the experiment, except in M3 and 4 where they again dominated the heterotrophic biomass at the end of the experiment (Fig. 8).

*Protoperidinium* spp. (Fig. 7B): *Protoperidinium* spp. peaked on day 4 in the warm mesocosm M4 with the highest magnitude, whereas it peaked on day 6 at slightly lower abundances in the other mesocosms. Thus, the timing and the magnitude of the maximum abundance were dependent on temperature. *Protoperidinium* spp. were not significant contributors to community biomass (Fig. 8).

### Microzooplankton groups from FlowCam analyses

*Gyrodinium spirale* (Fig. 7C): *Gyrodinium spirale* showed similar increases in abundances as the previous groups and peaked on day 5 in all mesocosms except in M1, where a less pronounced peak was found between day 6 and 8 (Fig. 7C). No significant differences were found between treatments, except for M1 *vs* M2 at day 5 (p<0.01; repeated measures ANOVA with Bonferroni *post hoc* test). In terms of specific biomass contribution to total heterotrophic biomass *G. spirale* showed a rather stable pattern in all mesocosms representing between 5 and 30% of the total biomass during the experiment (Fig. 8).

Ciliates (Fig. 7D): The abundance of ciliates peaked on day 5 in all mesocosms (Fig. 7d). The highest values were found in M2 (163±44 SD cells ml^−1^), followed by M4 (122±27 cells ml^−1^) and M3 (116±8 cells ml^−1^). It is notable that ciliate abundance and temporal patterns in the control mesocosm M1 were similar to the nutrient-amended ones (peak of 82±18 cells ml^−1^). In terms of biomass ciliates dominated the protozoan community during the phytoplankton peak period with decreasing biomass dominance toward the end of the experiment, reaching the lowest values at the end of the experiment in the increased temperature and CO_2_ treatments M3 and M4 (Fig. 8). Given the high variability between replicates we did not find significant differences between treatments (repeated measures ANOVA with Bonferroni *post hoc* test), except around the peak; i.e., day 5 (M1 *vs* M2) and day 4 (M2 *vs* M3).

### Protozoa community abundance descriptors and individual size evolution

We built a multiple-regression model (forward stepwise at p<0.05) for microzooplankton abundance throughout the study using the pooled data from all mesocosms and considering temperature, pH and all possible prey as descriptors (we excluded day 0 from the analysis as it was not influenced by any treatment). For *Gyrodinium spirale* the model explained 71% of the variance of *Gyrodinium spirale* based on Bacillariophyceae and Cryptophyta. (*Gyrodinium*. = 0.0015×Bacillariophyceae–0.0083×Cryptophyta+12. 85; p<0.001). The multiple-regression model for ciliates only showed significant values for Bacillariophyceae and pH. These two variables explained also 71% of the variation on ciliate abundance (ciliates = 45.5×pH+0.0027×Bacillariophyceae −355.3; p<0.001).

**Figure 9:**
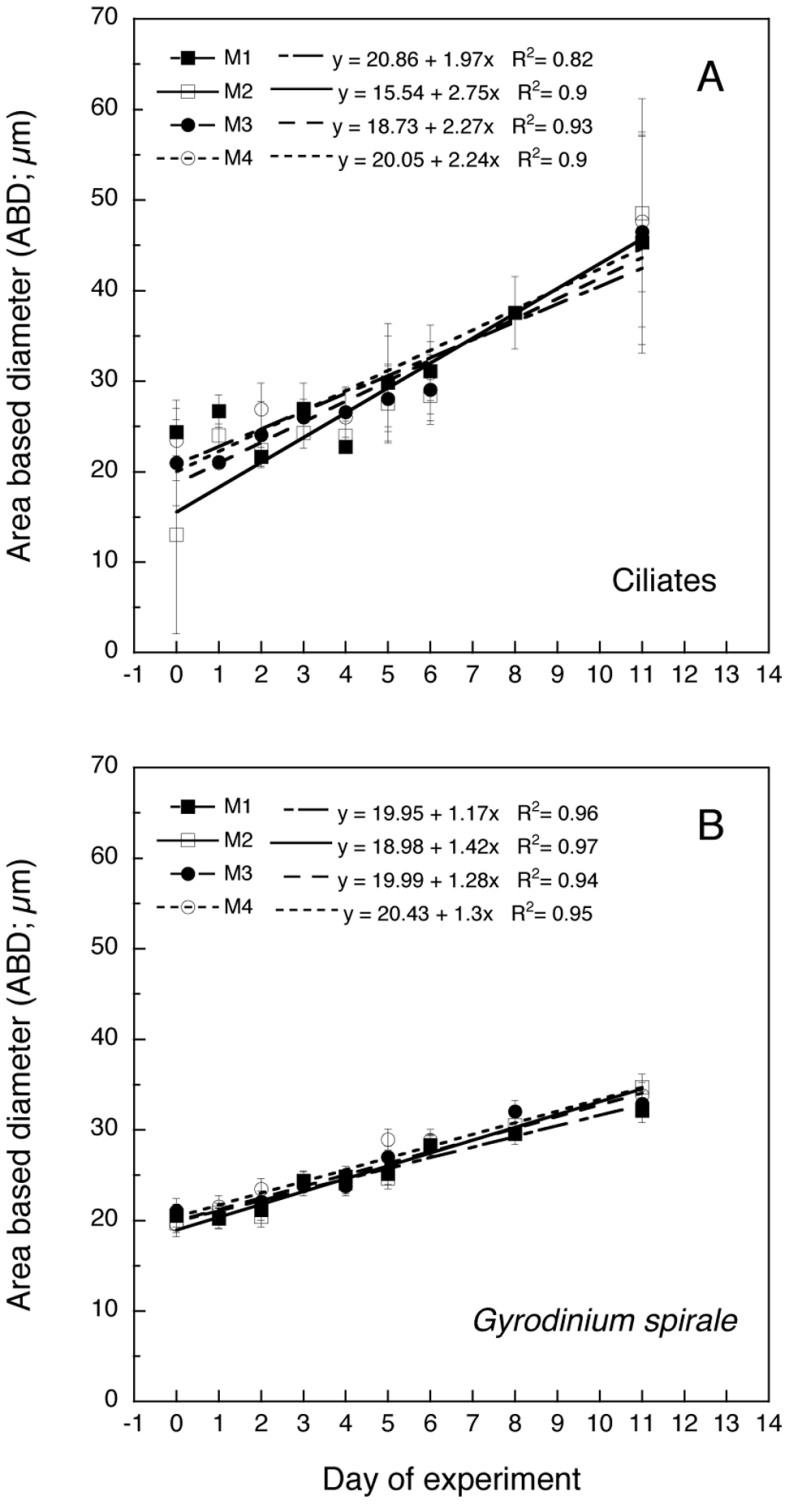
Mean cell size (area-based diameter, ABD) of two major heterotrophic groups (A: ciliates, B: *Gyrodinium spirale*). Regression models are given for each mesocosm treatment (M1-M4). Values are averages of the two replicates (A, B) for each treatment and error bars correspond to the SD.

There was a significant progressive increase in ciliate and *Gyrodinium spirale* mean cell size during the experiment (area-based diameter, ABD, Fig. 9), in all treatments. No significant difference was found between treatments in the slope of the linear relationships (ANCOVA test).

### Carbon budget and trophic efficiency

**Figure 10:**
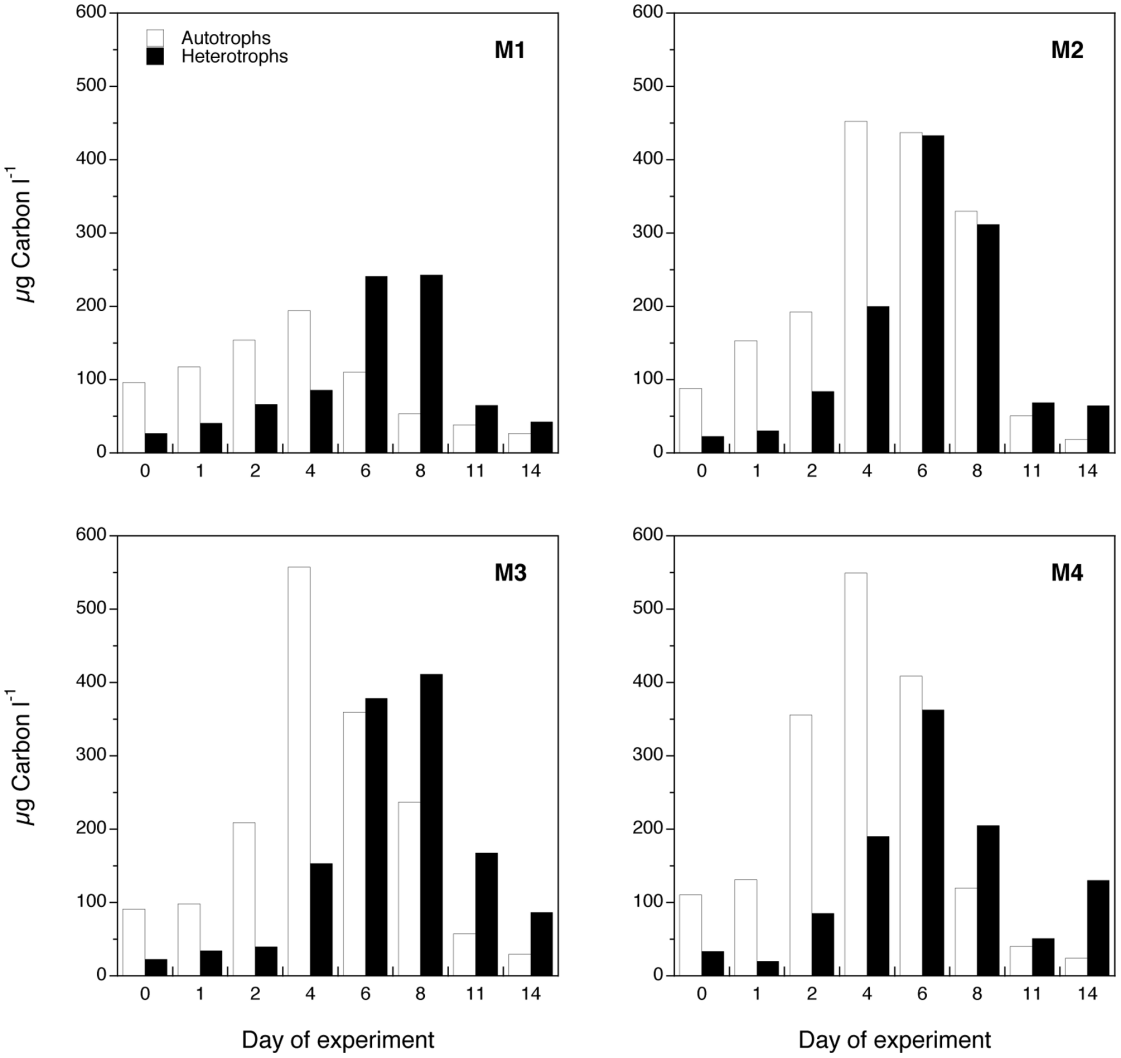
Autotrophic and micro-heterotrophic biomass (μg C l^-1^) in the four mesocosm treatments along the experiment. Values are averages of the two replicates (A, B) for each treatment and error bars correspond to the SD. See methods section for explanation on the biomass calculations.

The total biomass of autotrophs and protozoa over the time of the experiment (Fig. 10) showed that phytoplankton peaked before their grazers, the protozoans. The effect of enhanced nutrients on phytoplankton and protozoan (compare M1 with M2) resulted in a 2.2-fold increase of autotrophic peak biomass and 1.5-fold for heterotrophs. The effect of lowered pH (compare M2 with M3) indicated an increase in peak biomass of the autotrophs and a delayed peak timing of the heterotrophs. The effect of warming (compare M3 with M4) resulted in earlier onset and end of the phytoplankton bloom and faster breakdown of the protozoan peak in M4. Therefore, increased temperature seemed to accelerate both grazing and respiration of organic matter in these mesocosms. We can further explore the efficiency of the food web by calculating the quotient total protozoan (heterotrophic) / phytoplankton (autotrophic) carbon (H:A) after summing the carbon in each category (entire size spectrum for autotrophs and only protozoan microplankton for heterotrophs) for the duration of the experiment. The quotient H:A is proposed as a proxy for the trophic efficiency of the system [12]. Systems that support a higher biomass of heterotrophs per unit of primary producer are more efficient in their transport of energy towards upper levels of the food web. The quotient was the highest in the control (M1, H:A = 0.7±0.4 SD), intermediate at M2 and M3 (H:A equals 0.5±0.03 SD and 0.6±0.2 SD, respectively), and lowest at M4 (H:A = 0.4±0.2 SD).

### C:N ratio and food quality

**Figure 11:**
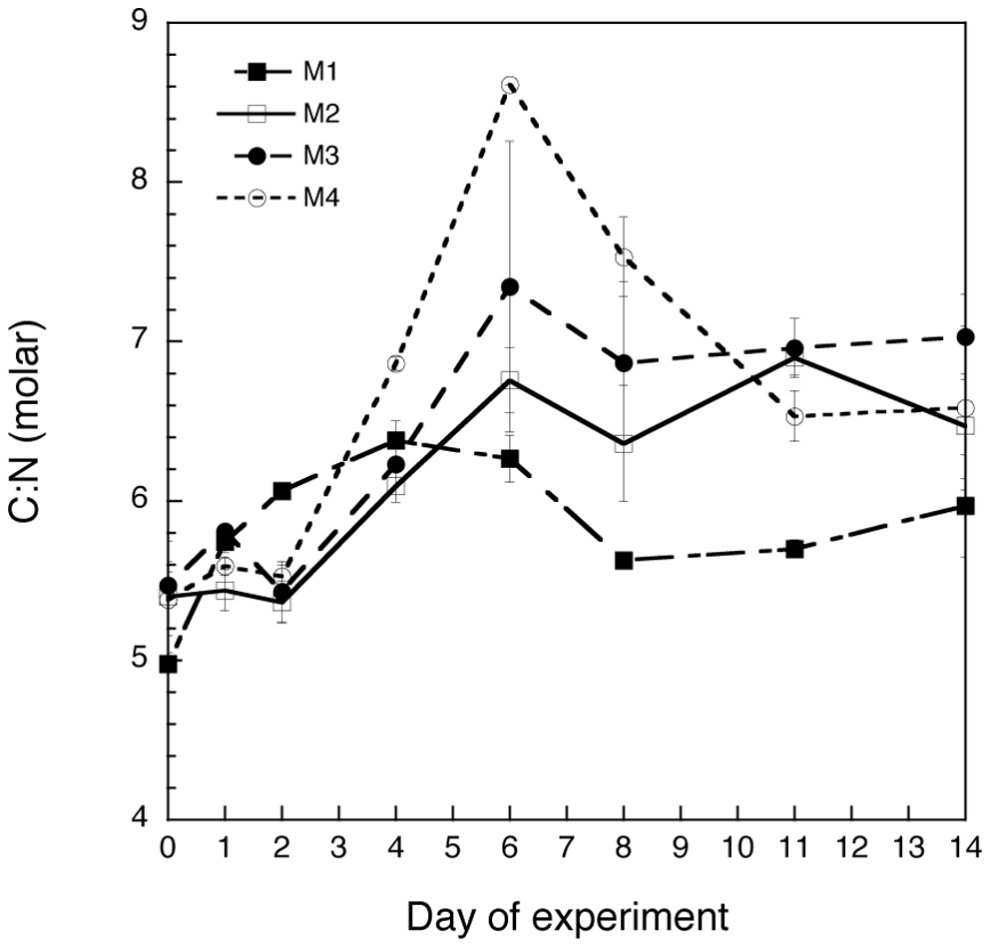
C:N molar ratios of the plankton size class of 2.7–10 μm in the four mesocosm treatments (M1-M4) throughout the experiment. Values are averages of the two replicates (A, B) for each treatment and error bars correspond to the SD.

The elemental composition of plankton in the size class of 2.7 to 10 μm changed during the experiment (Fig. 11). The molar particulate C:N ratio increased in all nutrient amended treatments until the phytoplankton peaked and either flattened out (M2 and M3) or decreased again (M4) towards the end of the experiment (p<0.01; two-way grouped ANOVA, factor: time). M1 showed only a small increase in C:N at the beginning and decreased slightly after day 4. M1 vs M2, and M2 vs. M3 showed significant differences in the development of the elemental composition of 2.7–10 μm sized plankton (p<0.05; two-way grouped ANOVA with repeated measures). Although M3 and M4 showed a similar overall shape of the curve, there was a significant difference of C:N ratios on day 6. The overall C:N values of M4 were the highest among treatments from day 4 to 8 reaching values above the Redfield ratio of 6.6∶1, indicating a poorer quality of the plankton during the phytoplankton peak period.

## Discussion

Mesocosms are a powerful experimental tool to address plankton food-web responses to climatic change. Certainly, mesocosms do not copy nature in all its details and they generally exclude the possibility of exploring possible long-term adaptation and evolution of marine plankton groups [46]. However, mesocosm experiments provide a more-realistic whole-ecosystem scale approach to address fast and mid-term complex community responses, compared to typical small-scale laboratory studies with limited species composition [14]. Another common criticism of mesocosm experiments is the lack of replication. Here, we use duplicated factorial treatments that allow for statistical analysis of the effects. Given the large amount of samples acquired during the experiment, and the negative effects of long-term preservation on these sorts of samples, we opted by a fast analysis (within the day of collection) of only one replicate per treatment. We are, nevertheless, confident regarding the accuracy of these data because they matched the patterns obtained by chl *a* analysis and by automated particle counters (FlowCAM). The latter measurements were performed on both replicates of each mesocosm and showed consistent responses among duplicates.

### Inorganic nutrient effects

As hypothesized (hypothesis *i*), the addition of inorganic nutrients resulted in phytoplankton bloom developments that were mainly dominated by the chain-forming diatom *Skeletonema marinoi*, a species that is commonly dominant during spring blooms in the fjord where the experiment was conducted. While the smallest and initially more abundant cells peaked and declined quickly, the chain-forming diatoms (also reflected in the >10 μm chl *a* fraction) still maintained a numerous population after the depletion of the measurable dissolved nutrients. This indicates that the diatoms were more efficient in utilizing the remaining nutrients, or had a larger storage of nutrients in their vacuoles [47]. However, we believe that most of the differences in the bloom breakdown dynamics of each group were related to microzooplankton selective grazing rather than to different nutrient recycling efficiencies. There are several observations that support this hypothesis. Parallel estimates of microzooplankton grazing using the dilution technique [48] revealed no significant grazing on large phytoplankton during the onset of the bloom, whereas grazing became very high during the peak period (Martínez unpublished). An inverse pattern was evident for small phytoplankton as the grazing pressure on these groups was strongest during the first days of the experiment (Martínez unpublished). These results are in agreement with the size-distribution of the dominant microbial grazers during each period. Small flagellates and small ciliates were dominant at the beginning coinciding with the smallest phytoplankton, and larger ciliates and dinoflagellates dominated later in the experiment coinciding with diatoms (Fig 9). Finally, the secondary peaks of re-mineralized silicate that appeared after day 8 (Fig. 2) suggest a strong grazing pressure on diatoms by microzooplankton (e.g., *Gyrodinium spirale*) at the end of the experiment.

As no nutrients were added to M1, recycling of nutrients was assumed to be important in this treatment. The same composition of species that flourished in the nutrient-amended treatments also peaked in M1, but to a lower extent and with a more even distribution among groups and a tendency to a higher relative contribution of heterotrophy. The communities that developed in the M1 mesocosms appeared to be of higher nutritional quality as indicated by lower C:N ratios. We thus conclude that these communities were more efficient in transferring matter toward higher trophic levels in the food web (heterotrophs) indicated by the disproportionate heterotrophic biomass compared to the autotrophic biomass (higher H:A quotient). As hypothesized (hypothesis *ii*), our data thus suggest lowered transfer efficiency in the planktonic food web under future short-term eutrophication scenarios. It seems that the extra autotrophic biomass in our nutrient-amended mesocosms mostly settled to the bottom of the mesocosms and was inaccessible to their potential pelagic grazers.

### Acidification

Acidification accelerated the development rate of the phytoplankton bloom as well as the composition of the microbial community (hypothesis *iii*). This effect was most noticeable in the diatom group, but was not as clearly manifested in calcifying algae such as *E. huxleyi* (compare e.g. [49]) or in total chl *a*. Diatoms, and especially *Skeletonema*, can grow exponentially at very low pH (<6.5), where other algae may experience growth limitation [50], [51]. High pH was shown to strongly reduce the NO_3_^–^ uptake rate as well as growth rates of *Skeletonema costatum* [50], [52]. Under non-nutrient-limited conditions, diatoms have a highly efficient carbon concentration mechanism (CCM) and RubisCO enzymes [53] and therefore appear better adapted to utilize the extra carbon provided by acidification, compared to non-CCM phytoplankton. This may explain the faster growth of the diatoms in the acidified mesocosms compared to the non-acidified ones [32]. We did not anticipate the faster breakdown of the phytoplankton bloom in the acidified treatments (M3) compared to M2 (only nutrient amended). Nutrient depletion seems not to have been the reason for the distinct dynamics of diatoms in the acidified *vs*. non-acidified treatments. Microzooplankton grazing may explain the faster decay of diatoms in M3 and M4, with ciliates and dinoflagellates being the dominant grazers during the early phase of breakdown of the bloom in M3 and M4, respectively (Martínez unpublished).

Chlorophyta were also stimulated by increased CO_2_ and reached higher concentrations at low pH. *Micromonas*-like phylotypes were the most abundant chlorophytes. They can possess two distinct types of CCM and have been shown to respond positively to low pH [54], [55]. Cryptophyta represented by *Plagioselmis prolonga* and *Chrysochromulina alifera* appeared to be differently impacted by acidification. While lower pH resulted in higher bloom concentrations of *P. prolonga* at ambient temperatures, this was reversed at increased temperatures. *C. alifera*, on the other hand, exhibited the opposite pattern. We cannot explain these responses, but suggest that effects of temperature may have outweighed any possible consequences of ocean acidification for these algae.

Acidification also affected certain protozoans, such as ciliates. The negative influence of lowering the pH on ciliates contradicts previous results on natural protozoan communities [33], [56], and could either be a direct consequence of pH on the physiology of the organisms or mediated by changes in their prey and/or predators. We are not aware of previous evidence indicating direct effects of pH on ciliates over the range we tested here. Coincidently with a lab study of some cultivated ciliates exposed to a pH of 7.8, close to our minimum of 7.7, Pedersen and Hansen [57] did not find a negative impact on the growth rates of ciliates. Results from a concurrent study (Dutz et al. unpublished) to this one show that the abundance of mesozooplankton, the common predators of ciliates, was low with 2–3 copepods l^−1^ at the phytoplankton peak, and 8–10 copepods L^−1^ at the end of the experiment, and that copepod abundances was not affected by any of the treatments. This further suggests that changes in the food source probably explain the detrimental effects of acidification on ciliates. However, acidification does not seem to have modified the bulk of prey for ciliates; the effects of this variable were manifested only on marginally-abundant groups. We do not have data for bacteria, but we may assume they follow a development similar to the 0.2–1 μm chl *a*. Also, the increase in ciliate size during the experiment points towards a gradual increase of the relevance of herbivory compared to bacterivory for this group [58]. We therefore suggest an indirect effect mediated by changes in prey biochemical composition (*i.e*., lowering on the food quality of prey; [24], [25]. Even though we did not detect significant differences in the C:N of seston in the M2 vs M3, acidification may still have caused changes in the fatty acid and / or amino acid composition, thus lowering the quality of the prey [24]. One could argue that this effect could be limited by the diversity of prey with different nutritional characteristics in the mesocosms [24], but our data suggest the opposite.

Despite the acidified mesocosms receiving extra inorganic carbon, the total organic carbon accumulated during the experiment was not different from the mesocosms with ambient pH. In other words, acidification *per se* seemed to impair carbon transfer to higher trophic levels, with the autotrophic peak enhancement not efficiently scaled up the food web, corroborating our initial hypothesis (*iv*).

### Temperature

Temperature, combined with acidification and eutrophication, had clear effects, both positive and negative on different planktonic groups. We observed a positive effect of future climate change conditions on Prymnesiophyceae, i.e. an earlier and more pronounced peak, and a negative effect, by the end of the experiment, on Cryptophyta [59]. Also in agreement with previous experimental studies, temperature caused an earlier breakdown of large diatoms, indicating an indirect effect of temperature-dependent mesozooplankton grazing [4], [60], [61].

Further, we focus on broader effects relevant for the overall trophic efficiency of this coastal planktonic ecosystem. Temperature is usually linked to physiology of organisms through the Q_10_ concept. Marine ciliates for example show a negative relationship of gross growth efficiency (GGE) and temperature [29]. This loss in efficiency in anabolic *vs.* catabolic processes is also apparent in terrestrial and marine autotrophs [62], [63]. This means that global warming can result in an imbalance between respiration and production where proportionally more carbon will be lost through respiration than assimilated by plants [62], [63]. Therefore, unless there is an evolutionary adaption, the initial direct consequences of increased temperature for plankton (increase of CO_2_ production) would be added to indirect impacts, such as reduction of autotrophic prey; both mechanisms resulting in an overall increase in CO_2_ in the ocean [33], [64].

Contrary to previous results [33], [65], [66], we did not detect a reduced time lag between prey and grazers with increased temperature. Instead we observed early decline of the bloom (assumed due to faster nutrient depletion combined with grazing), consistent with the model of Chen et al. [67], supporting higher grazing impacts due to global warming in eutrophic systems (hypothesis *v*). However, as hypothesized (hypothesis *vi*), because the negative relationship of protozoan GGE and temperature [29], this input of organic matter was not efficiently converted into new heterotrophic biomass but seems to have been respired or perhaps directed to smaller components of the microbial loop (prokaryotes) not considered here. Certainly, the rise of temperature of 3°C above an initial temperature of 12°C tested here, is not comparable to the one expected for polar ecosystems. In these systems the expected rise in temperature could have more profound consequences. However, for temperate and sub-polar climates we can anticipate an overall drop in the efficiency of shunting primary production to higher trophic levels (hypothesis *vii*). Nonetheless, these predictions should be understood in light of the future changes in the hydrodynamics of the area as well, which may be of higher relevance than the physiological changes *per se.*

### Conclusions

Focussing on single-stressors, such as acidification or temperature, in ecological research is unrealistic; it may approximate the response of the system to the chosen factor, but does not mimic expected climate change scenarios. Therefore, a multiple-stressor approach seems more appropriate. Our experimental set up did not include temperature as a single stressor because it is quite unlikely this factor can be detached from acidification in possible future scenarios and has been well studied already [26]. We investigated, therefore, the synergistic effects of eutrophication, acidification, and warming on a productive coastal plankton community. Our results also do not consider, or permit commentary, on possible long-term adaptations of organisms [32], [46], [68]. Moreover, there are variable results in the literature when testing climate change conditions on natural communities of other comparable productive areas (e.g., [18], [32], [33], [65], [66]). Hence, the results of each study may apply only to the particular area tested. Nevertheless, these investigations serve to establish a range of responses and to identify mechanisms that can pass unobserved in laboratory experimentation with single species. Ultimately, they may be crucial to improve parameterization of predictive models.

Under the conditions tested here, our research on fjord waters of western Norway suggest a shortening of the phytoplankton bloom period, with a slightly earlier timing in response to the predicted climate changes. These phenological changes should not matter much in systems where protozoans are the major grazers, because of their fast responses, short generation times and reduced growth limitation at low temperatures [4], [33], [65], [66], [69]. However, these changes may become critical for top consumers such as fish if intermediate metazoan grazers such as copepods “miss” the bloom [70]–[72]. Given that food quality for grazers may be affected by variation in prey species composition and by changes in the biochemical composition of the algal prey ([24], [25], [73], this study), we also expect a shift towards a more-autotrophic community and thus a less-efficient food web under future global change scenarios, especially when eutrophication is combined with acidification and warming in areas comparable to the investigated Norwegian fjord.
